# Long-term effects of intensive B cell depletion therapy in severe cases of IgG4-related disease with renal involvement

**DOI:** 10.1007/s12026-020-09163-3

**Published:** 2020-11-10

**Authors:** Giacomo Quattrocchio, Antonella Barreca, Andrea Demarchi, Roberta Fenoglio, Michela Ferro, Giulio Del Vecchio, Carlo Massara, Cristiana Rollino, Savino Sciascia, Dario Roccatello

**Affiliations:** 1 Nephrology and Dialysis Universitary Unit, and Center of Research of Immunopathology and Rare Diseases (CMID) San Giovanni Bosco Hospital, and Department of Clinical and Biological Sciences, University of Turin, Turin, Italy; 2 Department of Biomedical Sciences, University of Turin, Turin, Italy; 3 Pathology Unit, San Giovanni Bosco Hospital, Turin, Italy

**Keywords:** IgG4-related disease, IgG4-related disease with kidney involvement, Tubulointerstitial nephritis, Retroperitoneal fibrosis, Rituximab, B cell depletion therapy

## Abstract

IgG4-related disease (IgG4-RD) is an immune-mediated disorder often showing elevated serum IgG4 concentrations, dense T and B lymphocyte infiltration, and IgG4-positive plasma cells and storiform fibrosis. We prospectively evaluated for 4 years 5 patients with histologically proven IgG4-RD of whom 3 had tubulointerstitial nephritis (TIN) and 2 had retroperitoneal fibrosis (RPF). They received an intensive B depletion therapy with rituximab. The estimated glomerular filtration rate of TIN patients after 1 year increased from 9 to 24 ml/min per 1.73 m2. IgG/IgG4 dropped from 3236/665 to 706/51 mg/dl, C3/C4 went up from 49/6 to 99/27 mg/dl, and the IgG4-RD responder index fell from 10 to 1. CD20^+^ B cells decreased from 8.7 to 0.5%. A striking drop in interstitial plasma cell infiltrate as well as normalization of IgG4/IgG-positive plasma cells was observed at repeat biopsy. Both clinical and immunological improvement persisted over a 4-year follow-up. Treating these patients who were affected by aggressive IgG4-RD with renal involvement in an effort to induce a prolonged B cells depletion with IgG4 and cytokine production decrease resulted in a considerable rise in eGFR, with IgG4-RD RI normalization and a noteworthy improvement in clinical and histological features. Furthermore, the TIN subgroup was shown not to need for any maintenance therapy.

## Introduction

Often times, IgG4-related disease (IgG4-RD) is a heterogeneous disorder, whose features may include different organ involvement, high IgG4 concentration in the serum, and unusual histological findings. In detail, the latest include IgG4^+^ plasma-cell-enriched dense lymphoplasmacytic infiltrate, a storiform pattern of fibrosis, and obliterative phlebitis [1, 2].

Men in their 50s and above are most often affected and generally display non-severe clinical presentation, potentially mimicking several neoplastic, infectious, and inflammatory diseases [3].

Most common laboratory features are high serum concentration of IgG4 levels (observed in 50–70% cases) (> 135 mg/dl) often associated with hypergammaglobulinemia (80–90% of cases). Almost half of the patients also present with high serum IgE levels, peripheral eosinophilia, and hypocomplementemia. Less frequently antinuclear antibodies and rheumatoid factor can be observed [4–6].

Both T and B lymphocytes are involved in disease pathogenesis. Indeed, IgG4-RD lesions abound with activated clonally expanded cytotoxic CD4^+^ T cells and activated B cells including plasmablasts, and secrete cytokines such as IFN-γ and TGF-β, chemokines, growth factors, and enzymes, which recruit and activate fibroblasts thus being partially responsible for inflammation and fibrosis [7–13]. Clinically evident disease in one involved organ may be observed in up to 40% of patients, although it is not unusual to observe subjects in whom five or six organs are implicated [14]. About 15% of patients have kidney involvement [4–6, 11]. IgG4-related kidney disease (IgG4-RKD) includes a broad spectrum of features such as tubulointerstitial nephritis (TIN), membranous nephropathy (MN), pyelitis, and retroperitoneal fibrosis (RPF) with hydronephrosis [11, 15, 16]. IgG4-RKD patients, and particularly those with TIN, more often present with deeper hypocomplementemia and higher serum IgG4 levels than the other IgG4-RD patients [5, 15–18].

In patients with IgG4-related TIN, progressive kidney failure, proteinuria (often in sub-nephrotic range), leukocyturia, and mild hematuria can be observed [11, 16, 19–22], while IgG4-related RPF patients can present only with mildly elevated creatinine levels or normal renal function and normal urinalysis [16, 22, 23]. Contrast-enhanced computerized tomography (CT), magnetic resonance imaging (MRI), and ^18^F-fluorodeoxyglucose (FDG) positron emission tomography/computed tomography (PET/CT) are useful tools for diagnostic, staging, and monitoring purposes [22, 24]. The IgG4-RD responder index (RI) was recently established a reliable disease activity assessment tool for measuring response to therapy [25].

Patients usually respond favorably to the administration of glucocorticoids which are the mainstay of treatment for IgG4-RD [1, 3, 14, 22, 26] and for IgG4-RKD [11, 19–21, 27]. Nevertheless, not all patients achieve remission on glucocorticoid monotherapy even when given together with immunosuppressive agents [28]. Moreover, disease relapse rates following tapering or discontinuation are extremely high, and several adverse events are related to the long-term use of glucocorticoids [11, 12, 27, 29–31]. Rituximab (RTX) is used as well, but mainly as a second-line therapy both in refractory cases [32–35] and relapses and as maintenance therapy. Its use as induction therapy is limited [16, 36].

We have already described the encouraging outcome of a group of patients with severe systemic lupus erythematosus (SLE) who underwent intensive B cell depletion therapy (IBCDT) with a combination of 4 plus 2 infusions of RTX, cyclophosphamide, and methylprednisolone pulses [37]. Using this protocol in SLE patients, the additional immunosuppressive maintenance therapy to avoid disease relapse was not needed [38]. Studies on a larger series of lupus nephritis patients with a mean follow-up of 44.5 months confirmed the results [39]. Joining the fascinating debating on IgG4-RKD management, [40–42], we previously reported our pilot experience on the use of IBCDT in this condition [43]. Herewith, we aim to investigate the long-term safety and efficacy profile of the IBCDT, exploring the clinical, biochemical, radiological, and histological parameters of patients with severe IgG4-related TIN and IgG4-related RPF followed by our center for least 4 years after receiving IBCDT.

## Materials and methods

The cohort of patients with IgG4-related kidney disease examined in this study (Table 1) includes 4 males and 1 female, 3 with histologically proven IgG4-related TIN, and 2 with histologically proven IgG4-related RPF. Light microscopy and immunohistochemistry were used to examine renal and retroperitoneal tissue specimens. IgG4-related TIN was diagnosed according to Raissian’s criteria [17]. Deshpande’s consensus statement was applied for histological description [40]. The IgG4-RD diagnosis fits with the 2019 ACR/EULAR classification criteria [41], but we should acknowledge that they are not intended for diagnostic purposes.

**Table 1:** Clinical, histological, and laboratory features of patients

	Patient 1	Patient 2	Patient 3	Patient 4	Patient 5
Age (yrs)	74	70	82	54	73
Sex	Male	Male	Male	Male	Female
IgG4-RKD	TIN	TIN	TIN	RPF	RPF
Baseline eGFR (ml/min per 1.73m^2^)	11	8	8	48	63
12-month eGFR (ml/min per 1.73m^2^)	36	23	14	75	44
24-month eGFR (ml/min per 1.73m^2^)	37	22	19	75	49
36-month eGFR (ml/min per 1.73m^2^)	39	27	23	74	58
48-month eGFR (ml/min per 1.73m^2^)	32	27	26	66	62
Baseline sIgG (mg/dl)	2195	5201	2312	1034	1387
12-month sIgG (mg/dl)	721	826	571	839	372
24-month sIgG (mg/dl)	1026	821	695	618	739
36-month sIgG (mg/dl)	1421	898	596	652	557
48-month sIgG (mg/dl)	1480	715	763	756	778
Baseline sIgG4 (mg/dl)	354	1390	253	136	215
12-month sIgG4 (mg/dl)	39	80	36	70	N.A.
24-month sIgG4 (mg/dl)	215	37	17	42	19
36-month sIgG4 (mg/dl)	299	35	14	41	N.A.
48-month sIgG4 (mg/dl)	305	30	17	33	N.A.
Baseline sC3/C4 (mg/dl)	51/10	47/1	50/8	141/32	138/28
12-month sC3/C4 (mg/dl)	108/35	102/20	89/28	144/35	N.A.
24-month sC3/C4 (mg/dl)	130/56	120/25	90/30	117/29	123/30
36-month sC3/C4 (mg/dl)	95/27	100/18	90/28	136/31	N.A.
48-month sC3/C4 (mg/dl)	98/28	98/16	91/29	132/34	N.A.
Baseline IgG4-RD RI	12	9	9	6	6
12-month IgG4-RD RI	1	1	1	1	1
24-month IgG4-RD RI	1	1	1	3	1
36-month IgG4-RD RI	4	1	1	1	1
48-month IgG4-RD RI	3	1	1	3	1
Baseline CD20 (%)	10	3.1	13	6.6	10.8
12-month CD20 (%)	1.2	0.09	0.0	5	0.0
24-month CD20 (%)	1.2	0.08	4.4	0.0	5.6
36-month CD20 (%)	1.4	0.16	4.5	0.1	0.1
48-month CD20 (%)	1.8	0.3	4.8	2.7	0.0
Baseline Tregs (%)	9.0	5.4	N.A.	5.0	1.9
12-month Tregs (%)	3.0	2.5	2.2	5.5	1.0
24-month Tregs (%)	3.8	4.9	6.1	2.6	5.0
36-month Tregs (%)	4.9	5.5	N.A.	0.9	N.A.

*yrs*, years; *IgG4-RKD*, IgG4-related kidney disease; *TIN*, tubulointerstitial nephritis; *RPF*, retroperitoneal fibrosis; *eGFR*, estimated glomerular filtration rate; *sIgG*, serum IgG; *IgG4-RD RI*, IgG4-related disease responder index; *Tregs*, regulatory T cells; *N.A.*, not available

Patient N.1 had been diagnosed with a Sjogren-like syndrome that was being treated with small doses of oral prednisone. We admitted him to our unit 12 months later due to rapidly progressive renal failure. Kidney biopsy was in line with IgG4-related TIN.

Sixteen months prior to coming to our attention, patient N.2 had undergone mononephrectomy elsewhere since a neoplasm was suspected. Final histological diagnosis reported xanthogranulomatous pyelonephritis. Due to rapidly progressive renal failure, he was admitted to our unit where he underwent renal biopsy which revealed typical IgG4-related TIN.

On account of a malignant tumor, patient N.3 had been subjected to colon resection 20 years earlier. Admission to our unit was due to acute renal failure overlapping chronic impairment. Renal biopsy showed IgG4-related TIN.

TIN patients (N.1, N.2, and N.3) were investigated with ultrasonography at baseline and after 12 and 24 months. Contrast-enhanced CT was not performed due to the high risk of contrast-induced renal failure.

Patient N.4 was referred to our unit due to acute kidney injury. He also complained about lower back pain. Both ultrasound and CT imaging confirmed bilateral hydroureteronephrosis caused by retroperitoneal and periaortic abnormal tissue. Tissue specimens obtained by exploratory laparotomy revealed features of IgG4-related RPF.

Ten months prior to admission to our unit, patient N.5 had undergone radical hysteroannessiectomy on account of suspected uterine neoplasia with associated, at first monolateral, hydroureteronephrosis. Ureteral stents were implanted because of recurrent hydronephrosis caused by the presence of high metabolic activity in pathologic pelvic and periureteral tissue as detected at FDG-PET/CT. Surgical specimen re-examination following admission to our center confirmed IgG4-related RPF.

All patients underwent baseline and follow-up evaluation of the IgG4-RD RI, estimated GFR using the CKD-EPI formula, blood count, CRP, IgG/IgG4, C3/C4, CD20+ count, and urinalysis.

The 3 TIN patients had repeat percutaneous needle biopsies 12 months after the initial histological diagnosis. The two RPF patients underwent contrast-enhanced CT or MRI and FDG-PET/CT at baseline and during follow-up.

### B and T cell subset analysis

Circulating B and T cells in the peripheral blood were analyzed by flow cytometry. Whole blood samples collected in EDTA were stained with monoclonal antibodies against CD45 (APC 100 eBioscience Bender Medsystems, CA, USA), CD3 (FITC eBioscience Bender Medsystems, CA, USA), CD4 (PC7 Beckman Coulter, CA, USA), CD19 (Pacific Blue™, Beckman Coulter, CA, USA), CD20 (PE Beckman Coulter, CA, USA), CD25 (PerCP-eFluor 710 eBioscience/Bender Medsystems, CA, USA), and FOXP3 (PE Staining set, eBioscience Bender Medsystems, CA, USA).

### Renal and periaortic tissue pathology

Patients N.1, N.2, and N.3 underwent real-time ultrasound-guided percutaneous needle renal biopsies. Histological confirmation was acquired by open biopsy of the mass lesion in patient N.4 and by surgical hysteroannessiectomy in patient N.5. All tissue specimens were examined by light and immunofluorescence microscopy. Histological confirmation was obtained by immunostaining with anti-IgG and anti-IgG4 antibodies in every case.

### Treatment protocol

The IBCDT protocol we previously described for the treatment of severe cases of SLE with nephritis was followed for the treatment of [37, 39]. Three pulses of 15 mg/kg methylprednisolone were administered intravenously. Afterward, oral prednisone (0.8 mg/kg/day, gradually tapered until discontinuation over 4 months) *plus* (patients N.1, N.2, N.4, and N.5) 2 pulses of 500–750 mg cyclophosphamide (on days 1 and 15) *plus* 4 weekly rituximab administrations (375 mg/m^2^) were delivered. Two more doses of rituximab were given 1 and 2 months after the last weekly infusion.

We did not administer cyclophosphamide to patient N.3 on account of his prior history of colon cancer.

Patient N.5 received a maintenance regimen consisting of RTX 500 mg every 6 months for 18 months, followed by RTX 500 mg after 12 months.

Patients N.2 and N.5 received isoniazid prophylaxis on account of positive QuantiFERON-TB Gold test results.

### Statistical analysis

For the comparison of variables at baseline and follow-up, Student’s *t* test was used for normally distributed parameters and the non-parametric Mann-Whitney test for non-normally distributed parameters. For these analyses, the SPSS (IBM Corporation, NY, USA) software was used. *P* < 0.05 was considered statistically significant.

## Results

**Fig. 1:**
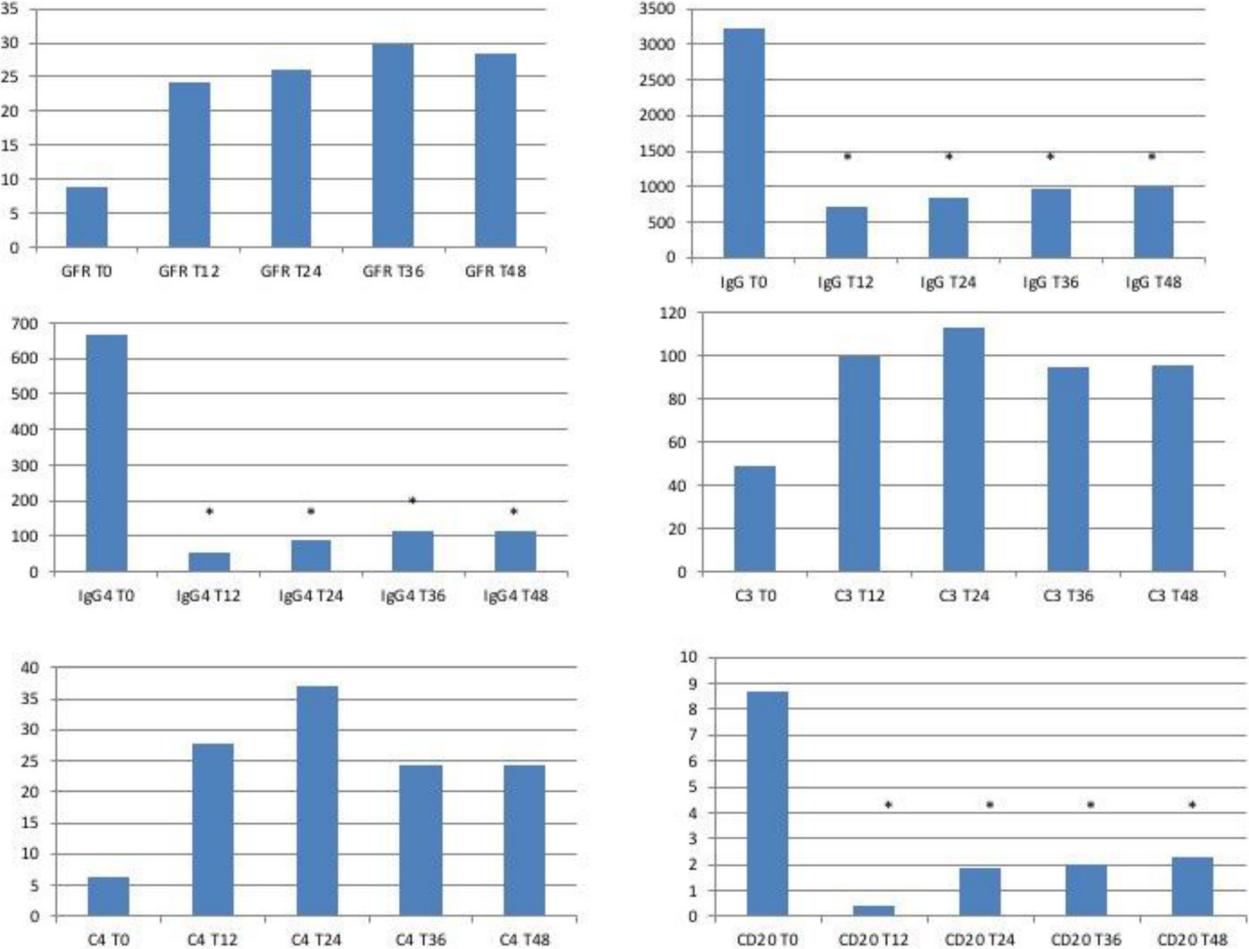
Laboratory parameters. GFR, IgG, IgG4, C3, C4, % of CD20+ at T0 and yearly up to 4 years of follow-up. eGFR, glomerular filtration rate (ml/min); CD20+ are expressed as % of total lymphocytes; T, time as expressed in months

Characteristics at inclusion and during the 48 month follow-up are presented in Table 1 and Fig. 1.

Kidney function substantially improved in the 3 TIN patients (N.1, N.2, and N.3) who underwent IBCDT. eGFR at 12 months had increased from 9 (range, 8–11) to 24 (range, 14–36) ml/min per 1.73 m^2^. This trend toward amelioration continued at 24 months (26 ml/min per 1.73 m^2^, range, 19–37), at 36 months (29 ml/min per 1.73 m^2^, range, 23–39), and at 48 months (28 ml/min per 1.73 m^2^, range, 26–32). Patient N.4 also showed an improvement in eGFR values at 12, 24, 36, and 48 months (from 48 to 66–75 ml/min per 1.73 m^2^). The eGFR values of patient N.5 (RPF with bilateral ureteral stent implantation) remained stable 3 years after the stents had been explanted.

Definitive improvement of the immunologic parameters was observed for TIN patients (N.1, N.2, and N.3), and especially with regard to total serum IgG, which dropped from 3,2 (range, 2,2–5,2) to 706 (range, 571–826) mg/dl at 1 year, and to 986 (range, 715–1480) mg/dl at 4 years. IgG4 subclass also decreased from 665 (range, 253–1390) to 51 (range, 36–80) mg/dl at 1 year and to 117 (range, 17–305) mg/dl at 4 years. Complement mean level rose to normal. C3 increased from 49 (range, 47–51) to 99 (range, 89–108) mg/dl at 1 year and to 95 (range, 91–98) mg/dl at 4 years, while C4 went from 6 (range, 1–10) to 27 (range, 20–35) mg/dl at 1 year to 24 (range, 16–29) mg/dl at 4 years. A drop in total IgG and IgG4 levels was also seen in our RPF patients (N.4 and N.5) on account of immunosuppressive treatment. C3 and C4 levels, which were not deranged at baseline, remained unchanged throughout follow-up. Urinalysis revealed mild proteinuria and mild microscopic hematuria at baseline, all of which rapidly and persistently improved after treatment (data not shown). All five patients had isolated kidney involvement requiring immediate treatment, except patient N.1 who presented also moderate salivary gland disease. The 3 TIN patients had increased serum IgG4 concentration at baseline. The IgG4-RD RI decreased from 8.4 (range, 6–12) to 1 at 1 year and remained stable during follow-up. The exceptions were patient N.1 whose RI rose to 4 after 36 months related to a moderate rise in IgG4 levels and patient N.4 whose RI increased to 3 after 24 and 48 months because of disease flares.

Pre-treatment CD20^+^ B cell mean values dropped from 8.7 (range, 3.1–13) to 0% within 7 days of the 4th infusion (data not shown), mildly increased to 1.2% (range, 0–5) 12–15 months later, and rose to 2.2% (range, 0–5.6) after 2 years.

### Histological features of IgG4-RKD patients

**Fig. 2:**
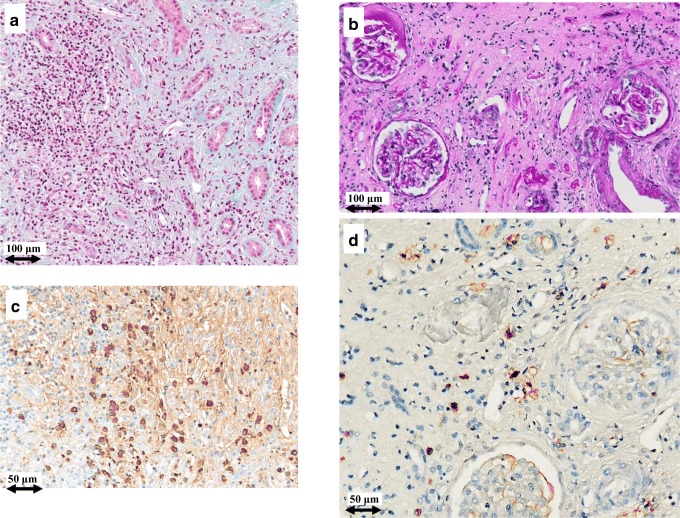
Light microscopy and immunohistochemistry findings in patient #1. Plasma cell infiltrate before (**a**) and after (**b**) intensive B cell depletion therapy (IBCDT). IgG^+^ plasma cells before (**C**) and after (**D**) IBCDT. **a** Masson’s trichrome (original magnification × 100). **b** Periodic acid-Schiff solution (PAS) (original magnification × 100). **c**, **d** Immunohistochemical staining (original magnification × 200)

**Fig. 3:**
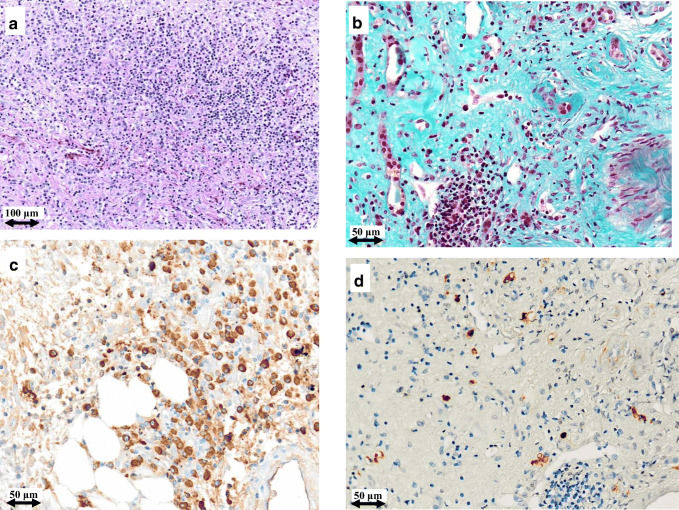
Light microscopy and immunohistochemistry findings in patient #2. Plasma cell infiltrate and storiform fibrosis before (**a**) and after (**b**) intensive B cell depletion therapy (IBCDT). IgG^+^ plasma cells before (**c**) and after (**d**) IBCDT. **a** Periodic acid-Schiff solution (PAS) (original magnification × 100). **b** Masson’s trichrome (original magnification × 200). **c**, **d** Immunohistochemical staining (original magnification × 200)

A repeat renal biopsy was carried out on TIN patients (N.1, N.2, and N.3) 1 year after the first procedure. Representative results (patients N.1, N.2) are presented in Figs. 2 and 3. A noteworthy decrease in interstitial plasma cell infiltrates could be appreciated by light microscopy study. Immunohistochemistry revealed IgG4/IgG-positive plasma cell normalization (from 40 to 4% and from 60 to 2%, respectively). Similar histological improvement was observed in patient N.3 (not shown). In particular, the first biopsy in patient 1 demonstrated a more densely cellular inflammatory lesion with expansile storiform interstitial fibrosis in about 80% of the evaluated sample, while in the second biopsy, we observed a marked reduction of storiform interstitial fibrosis that involved only about 5% of the examined material with a prominent reduction of the inflammatory plasmacellular component (from 45 plasma cells/hpf to 7/hpf). Furthermore, immunofluorescence from the first biopsy showed granular immune deposits (IgG, IgM, and C3) in the tubular basement membrane which turned out negative in the second sample. In patient 2, the interstitial fibrosis remained substantially identical between the two samples, whereas there was a significant reduction of plasma cells (from 70 to 90/hpf to scattered plasma cells/hpf).

**Fig. 4:**
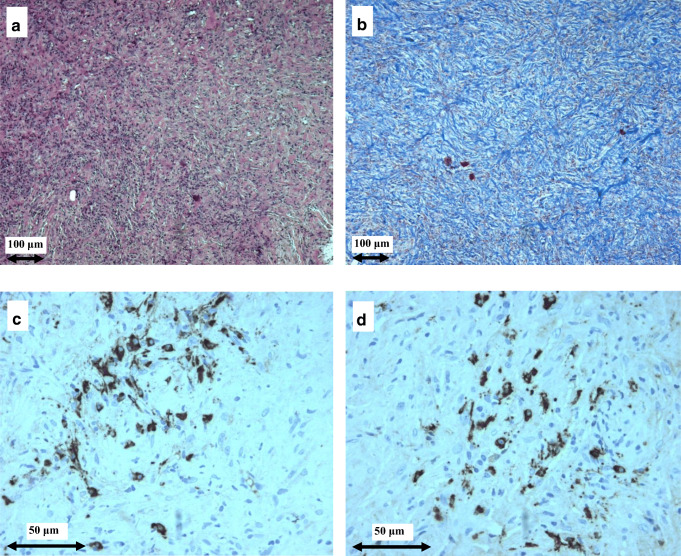
Light microscopy and immunohistochemistry findings in patient #4. Dense plasma cell infiltrate (**a**) and storiform fibrosis (**b**) in surgical periaortic tissue biopsy, typical of IgG4-related retroperitoneal fibrosis. IgG^+^ plasma cells (**c**), and IgG4^+^ plasma cells (**d**). **a** Hematoxylin and eosin (original magnification × 100). **b** Masson’s trichrome (original magnification × 100). **c**, **d** Immunohistochemical staining (original magnification × 400)

Light microscopy and immunohistochemistry results of the surgical periaortic tissue biopsy carried out on patient N.4 are shown in Fig. 4. Dense lymphoplasmacytic infiltrate could be observed, as could the typical storiform pattern of fibrosis and IgG4^+^ plasma cells (> 30/HPF).

### Imaging aspects of IgG4-RKD patients

Ultrasonography in patients N.1, N.2, and N.3 showed no definite atrophic change after 12 and 24 months.

**Fig. 5:**
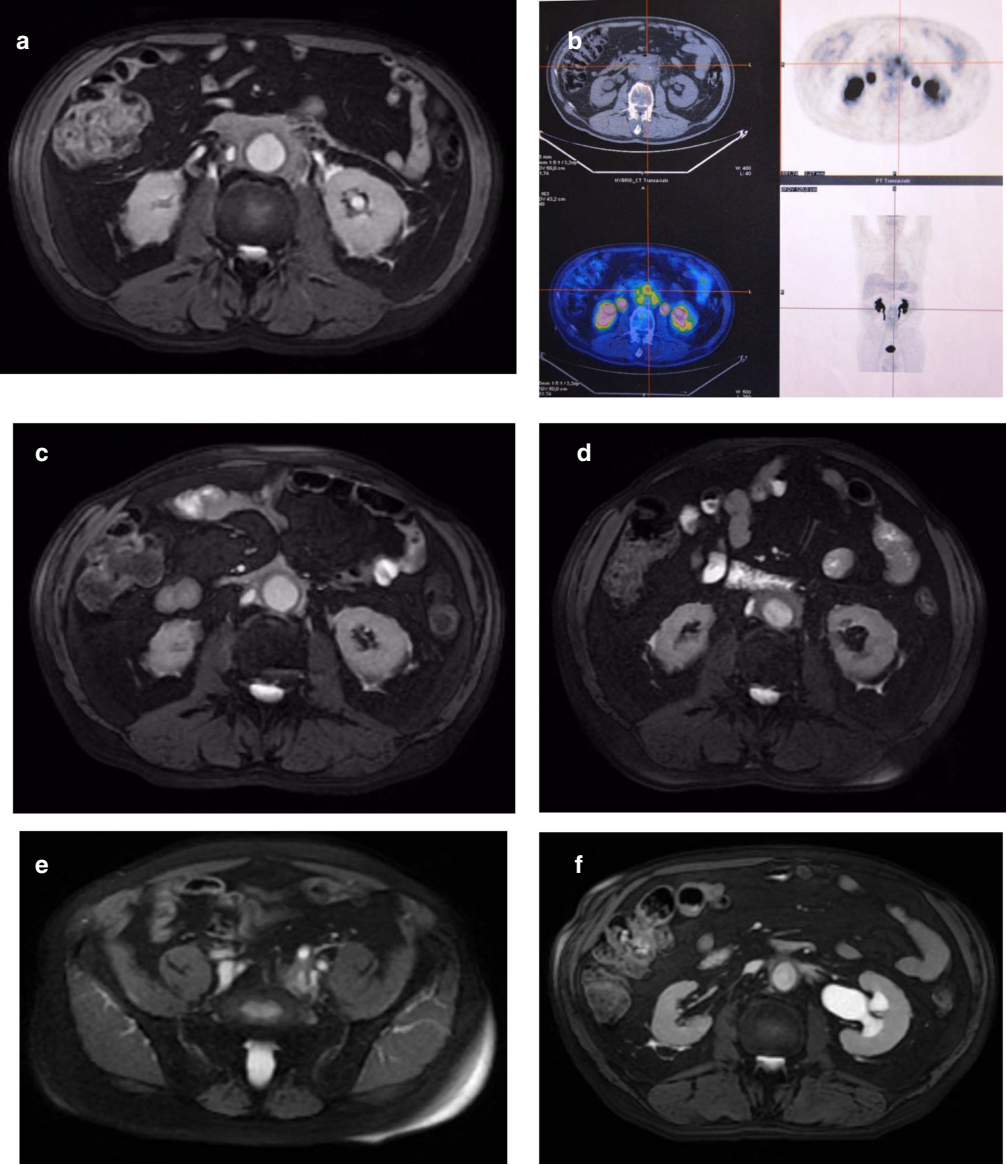
Radiologic features of patient #4. Magnetic resonance and PET/CT imaging before (**a**, **b**), at 5 months (**c**), at 16 months (**d**) after treatment and at 4 years (**e**, **f**): **a** and **b** abnormal periaortic soft tissue, axial diameter 17 mm, showing high metabolic activity; **c** 60% reduction of tissue axial thickness; **d** 80% increase in periaortic mass axial diameter; **e** appearance of abnormal soft tissue around the left iliac vessels and left ureter; **f** left hydronephrosis

Changes in the retroperitoneal, periaortic tissue of patient N.4 are shown in Fig. 5*.*

Patient N.4 relapsed 16 months later concomitantly with a circulating CD20-positive cells increase, and was treated with oral steroids (tapered over the next 3 months), as well as RTX 375 mg/m^2^ every 4 months for 12 months, and 500 mg every 6 months for further 12 months. MRI and PET/CT scans revealed a gradual decrease in retroperitoneal tissue. Twelve months following the last RTX infusion, MRI and PET/CT studies highlighted left hydronephrosis caused by a pelvic fibro-inflammatory mass. The patient received intravenous steroids and RTX 375 mg/m^2^ every 6 months, which resulted in complete remission.

**Fig. 6:**
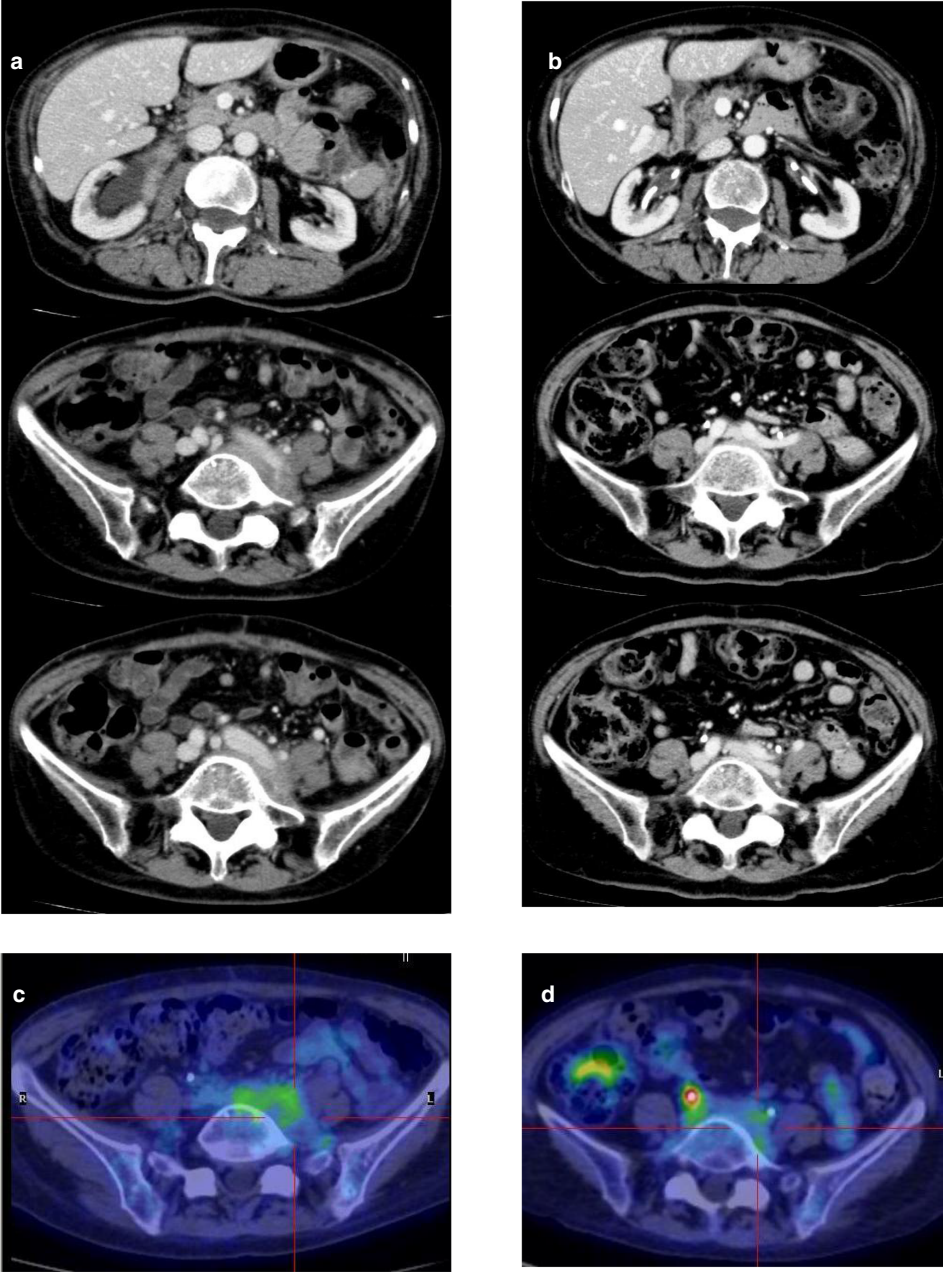
Radiologic features of patient #5. CT scan and PET/CT imaging before starting therapy (**a**, **c**) and at 6 months (**b**, **d**): **a** retroperitoneal and perivascular fibro-inflammatory tissue, with (**c**) high metabolic activity; **b** complete disappearance of abnormal tissue, with (**d**) persistence of residual minimal pelvic metabolic activity

Radiologic imaging carried out on patient N.5 prior to beginning the IBCDT protocol and again at 6 months is depicted in Fig. 6**.** Retroperitoneal and perivascular fibro-inflammatory tissue on CT scan completely disappeared although residual minimal pelvic metabolic activity was still visible on PET/CT scans. The ureteral stents were explanted after 14 months, and no evidence of hydronephrosis was seen in the following annual MRI.

### Treatment-related complications of IgG4-RKD patients

Transient insulin therapy was administered to patients N.1 and N.2 in whom a steroid-induced diabetes mellitus occurred. Insulin administration was discontinued after the treatment.

While on immunosuppressive therapy, patient N.4 developed mild, acute bronchopneumonia which was quickly and thoroughly brought under control with oral antibiotics.

## Discussion

Glucocorticoids are the mainstay of treatment for both IgG4-RD [1, 3, 14, 26] and IgG4-RKD [11, 19–21, 26, 27].

An initial prednisolone dose of 0.6–1.0 mg/kg/daily, gradually tapered over 2–4 weeks based on clinical response, is the standard approach for IgG4-RD patients, especially those with type 1 autoimmune pancreatitis. Improvements in both clinical and laboratory findings are often quick, though there are remarkable variations depending on which organ is affected and the degree of fibrosis [1, 2, 12]. Despite initial response, tapering or discontinuation of steroid administration often results in high relapse rates [11, 12, 14, 22, 28, 29]. Since patients receiving steroids are susceptible to several adverse effects, conventional immunosuppressants (e.g., cyclophosphamide, mycophenolate mofetil, methotrexate, azathioprine) have been administered either as glucocorticoid-sparing agents or to treat patients showing incomplete response. Nonetheless, the clinical utility of these agents remains to be proved [2, 12, 14, 30].

The efficacy of RTX in ameliorating the clinical and serological features of IgG4-RD patients with active inflammation [16, 26, 27, 32, 33], even without steroid administration [34], has been shown in about two hundred patients recruited in retrospective or uncontrolled non-randomized studies. RTX administration lowers serum IgG4 and maintains serum IgG4 concentrations low and the disease quiescent even after B cell reconstitution [33]. The efficacy of maintenance therapy with RTX has been proven as well [30, 36].

Most patients with IgG4-related TIN [1, 17, 20, 27, 35] and some patients with IgG4-related RPF [14, 24, 31] actually benefit from steroid therapy. Nevertheless, renal function recovery may not be complete, and renal cortical atrophy may develop despite glucocorticoid therapy, particularly in patients presenting pre-treatment renal insufficiency and serum IgE elevation [42]. Moreover, after tapering or discontinuing steroid administration, renal dysfunction often relapses and may lead to irreversible kidney failure, particularly in those who already have advanced renal damage [11, 21, 27]. As compared to glucocorticosteroids, a more effective and long-lasting response might be achieved when using RTX in IgG4-RKD patients [36]. This aspect has not been investigated thoroughly. Besides our experience (ref. 16, 43, and present data) and a recent report on IgG4-related aggressive TIN [44], IgG4-RKD patients have not been specifically identified as an ideal target to be given RTX.

Recommendations for managing IgG4-RD were recently drawn up by an international panel of experts. They confirmed the use of glucocorticoids as first-line drugs for remission induction in all patients with active, untreated disease (94% agreement). On the contrary, there was less agreement (46%) with regard to the administration of steroid-sparing agents such as RTX [45]. This may be related to a limited gap between the results that were observed when administering steroids compared to RTX monotherapy.

More attention should be reserved to the most severe cases who are highly susceptible to getting worst outcome. These cases specifically include IgG4-related TIN (in which RTX might be indicated as a single course) and especially RPF (in which a maintenance RTX regimen after the initial course is probably to be recommended).

We applied the RTX-based regimen that we follow in patients with severe SLE nephritis [37, 39]. The rationale of the combination therapy with glucocorticoids and cyclophosphamide relies on both the synergic effects of RTX on CD19-positive cells and on broadening immunomodulation to a wider spectrum of T and B cells, which would therefore selectively modulate different B cell subpopulations, and inhibit B cell antigen-presenting cell function, T cell-B cell interactions, and eventually fibrogenesis [46, 47].

IgG4-related TIN patients displaying greatly reduced eGFR and extremely high IgG4-RD RI at presentation (mean 10, range 9–12) responded remarkably well with regard to both renal function and immunologic parameters including the normalization of circulating regulatory T cells at the end of therapy. This observation suggests a potential role for these cells in the disease pathogenesis [7] and could represent a laboratory marker in the follow-up of patients. Moreover, they achieved near complete normalization of IgG4-RD RI.

Regarding radiological findings, even if we could not properly investigate these patients with contrast-enhanced CT due to their severe renal failure, it is not excluded that also the development of renal atrophy reported with steroid therapy [42] could have been attenuated by IBCDT. Notwithstanding the severity of the disease in our series, and despite administration of no further immunosuppressant therapy, after 4 years, these benefits still persist, confirming the efficacy of IBCDT in IgG4-RKD previously reported by our group [43] and extending the observation to 48 months of follow-up. This is in line with previously reported results regarding severe SLE patients with long-term follow-up (44 months) [39]. Moreover, as previously reported by others [48, 49], very limited residual IgG-positive and total plasma cell infiltration was observed in the repeat renal biopsies that were carried out 12 months following presentation (i.e., about 8 months after completely discontinuing therapy). This further upholds the long-term effects of IBCDT. It is noteworthy that as far as interstitial fibrosis is concerned, the peculiar storiform pattern characterizing IgG-RD was not evident any more.

Concerning IgG4-related RPF, characterized by a high recurrence rate [14, 36, 50], our data confirmed a maintenance immunosuppressive therapy to be appropriate. When examining the clinical course of patient N.4, a relapse at 16 months and again at 4 years despite the encouraging initial drop in the IgG4-RD RI was observed. That confirms the need for MRI or PET/CT monitoring at 6- to 12-month intervals in order to quickly identify and treat disease reactivation.

This study has a main limitation in the number of subjects. Furthermore, patient N.3 did not receive cyclophosphamide, and patient N.5 received RTX as maintenance treatment for the aforementioned reasons, so the treatment protocol was not the same for all the patients. However, severe renal involvement is quite unusual in IgG4-RD (which itself is per se a very rare disease), and the treatment should be personalized to each patient, thus making the feasibility of a randomized controlled study, at least in this restricted sample of patients, unrealistic.

The main strengths of this study consist in the long follow-up and, most importantly, the histological monitoring through repeat renal biopsies in TIN patients. Ultrasonography imaging after 12 and 24 months in our patients was unchanged; thus, it could not adequately inform about the actual disease evolution. Re-examining both histology and therapy 1 year post-diagnosis proved to be useful for directing further therapeutic decisions, providing insight into possible disease progression, and, as with our subjects, supporting therapy discontinuation.

In conclusion, while if untreated IgG4-RKD can lead to end-stage renal failure and steroid treatment may not suffice when dealing with the most serious forms of IgG4-TIN and is constrained by numerous side effects, a single course of IBCDT was found to be safe and effective in the long term in IgG4-TIN. Subjects with IgG-RPF need maintenance treatment and close follow-up.
